# Continuous Glucose Monitoring and Glycemic Control in an Adult Without Diabetes: Over 4,000 Automated Recordings Guide Contingency-Shaped Learning

**DOI:** 10.1177/26924366251380125

**Published:** 2025-09-15

**Authors:** David S. Black, My. H. Vu, Alaina Vidmar, Braden Barnett

**Affiliations:** ^1^ Department of Population and Public Health Sciences, Keck School of Medicine, University of Southern California, Los Angeles, California, USA.; ^2^ Biostatistics and Data Management Core, The Saban Institute, Children’s Hospital Los Angeles, Los Angeles, California, USA.; ^3^ Department of Pediatrics, Division of Pediatric Endocrinology, Children’s Hospital Los Angeles and Keck School of Medicine of USC, Los Angeles, California, USA.; ^4^ Division of Endocrinology and Diabetes, Keck School of Medicine, University of Southern California, Los Angeles, California, USA.

**Keywords:** CGM, feedback, digital health, glycemic control, glucose, without diabetes

## Abstract

The role of continuous glucose monitoring (CGM) in glycemic control among individuals without diabetes is not well understood. Specifically, the feedback generated may serve as a promising technology for obesity and lifestyle management among people without diabetes. In this case study, we used a CGM system to continuously measure sensor glucose levels in an adult female with BMI >30 without diabetes, capturing over 4,000 automated sensor recordings. The participant received real-time glucose readings via a smartphone app, which displayed values in a line graph. A shaded area indicated glucose levels exceeding the upper normoglycemic range, and an audio alert was triggered for high excursions (>140 mg/dL). We analyzed the percentage of daily time out of range (%TOR) across 16 days and evaluated whether standalone CGM feedback could reduce %TOR over the wear period. The participant reported checking the app multiple times per day every day. A notable reduction in average daily %TOR was observed, decreasing from 9.2% in the first sensor phase to 1.9% in the second. The median daily number of high-glucose excursions declined from 1.5 to 0.0. These findings suggest that standalone CGM offers feedback associated with glycemic control and can bring daily %TOR to under the recommended target of 5% in an individual with a metabolic risk but without diabetes. CGM may play a key role in obesity and lifestyle management by linking glucose tracking with behavior modification strategies, amplifying feedback necessary for contingency-shaped problem solving.

## Introduction

Humans inherit a capacity to respond variably in environments that generate consequences following the enactment of alterations in behavior. Those consequences can yield observable feedback generated from the behavior and can strengthen self-modification. Self-generated feedback variables that reveal health status can operate to elicit change in an individual’s response type and frequency that serves to avoid negative health consequences. Common problem-solving responses when feedback is presented can include self-managing variables involved in health status. Each self-control response can yield observable changes, and those that bring the individual closer to solving the health problem at hand are considered reinforcing.^1^ For example, when a person modifies diet then observes a post-meal increase in glycemic control, the problem is observed as moving closer to resolution in relation to a goal, increasing the probability of similar dietary changes and glucose self-monitoring in the future of that individual.

What does problem-solving entail for glycemic control? Problem-solving is any intentional behavior used to modify a different aspect of future behavior, thereby bringing a problem under greater control. Thus, an individual may wear a continuous glucose monitor (CGM) sensor and view glucose feedback continuously to modify future dietary behaviors that lower glucose levels.^2^ Effective problem-solving typically hinges on the strength of the stimulus generated.^3^ By continuously tracking glucose levels with a CGM system as the approach to generating continuous feedback, a user relies on a digital tool to create a stimulus to determine whether their new responses are effectively solving the problem. For example, a person may visually detect a glucose excursion in response to a high alert following select meals and make meal type and timing modifications accordingly.^4,5^

Two operations explain how humans solve problems.^1^ The first is *rule-following*. People may follow the verbal or textual guidelines offered by a trusted professional, which reveal the contingencies involved in the health problem based on associations historically identified from other cases with similar health problems. Common instructions in the case of obesity include advice to modify diet, increase physical activity, and adhere to medical checkups.^6^ Health instruction does not typically involve the patient’s direct experience of the behavior–consequence contingencies as they unfold. The second operation is *contingency-shaped*. A person responds with a new type and/or frequency of behavior because the consequence of response is directly experienced, and the behavioral modifications can thus be reinforced by feedback. By viewing the changing value of a feedback stimulus pertinent to modifiable variables, a person can derive their own rules for self-control geared toward solving a problem. For example, a person gains direct experience of the operating behavior–consequence contingencies, and the shaping of behavior occurs by glucose values within range as a reinforcing stimulus generated by modifications in behavior.

The role of a standalone CGM system with feedback to inform behavior changes that move glucose levels within target range is not thoroughly studied in adults without diabetes.^7^ In this case study, we aim to advance this foundational knowledge by capturing glucose levels by a CGM system in an adult female with BMI >30 who received instructions on the CGM system and the definition of blood glucose and the target level of glycemic control. We analyzed sensor glucose levels across 16 days, during which she received continuous feedback on her personal smartphone in the form of a graphical display, with a dotted line indicating time on the x-axis and glucose levels on the y-axis. A shaded gray area highlighted periods when glucose levels exceeded the set high range and alerts chimed at high range episodes only. Using automatically recorded glucose levels in milligrams per deciliter (mg/dL) every 5 min, we tested whether CGM feedback promotes glycemic control across the wear period. The contribution of this investigation is to determine whether and by how much standalone CGM feedback increases glycemic control in a case with a metabolic risk but without diabetes. Findings hold the potential for social validity such that meta-analyses have identified obesity versus normal weight as generating a sevenfold increase in diabetes risk.^8^

## Method

### Design

We conducted a continuous measurement prospective case study in March 2024. A Dexcom CGM (G6 Pro, 2024 Dexcom, Inc.) was self-applied on the upper back of the preferred arm. The pilot participant was instructed on how to use the Dexcom app on their smartphone. She was instructed to wear the CGM for 20 days, with a replacement of sensor at day 10, and to check the app as many times as desired and in response to auditory chimes made by the app at glucose excursions >140 mg/dL. All protocols were approved by the Institutional Review Board of the affiliated university (UP-23-01001) and the associated trial is registered with ClinicalTrials.gov (NCT06472297). Our reporting practices follow the systematic coding of observed human behaviour checklist recommendations.^9^

### Measurement

A computer-based survey was used to capture demographic and centers for disease control and prevention (CDC) Prediabetes Risk Test^10^ scores, and fingerprick Hemoglobin A1c% was captured at baseline to confirm a nondiabetes case. CGM data were downloaded from the Dexcom Clarity server (2024 Dexcom, Inc.). Data collection began when a sensor was placed and paired to the Dexcom app and ended when the sensor was removed. The study used individual glucose values and the standardized glycemic variability metrics for each sensor wear period. Metrics included mean glucose, %CGM wear time, %time >140 mg/dL (minutes spent over 140 mg/dL divided by total minutes of CGM analytic wear time), excursions >140 mg/dL, and % excursion peaks >140 mg/dL. For interpretation, the guideline is that percent time out of range (%TOR) >140 should be <5% for adults without diabetes.^11^

### Analyses

To ensure valid data, analyses excluded data points from the first day of any sensor placement, the day of sensor replacement, and the sensor removal day. These start and end days commonly generate false patterns of variability, often considered a calibration period. Glucose values for daily 24-h periods and daily aggregated glucose scores for the two sensor periods were generated, with glucose <140 mg/dL being the target criterion. No minimum glucose target was included. Daily standardized glycemic variability (GV) metrics were calculated using the R package *cgmanalysis* and summarized as median (Interquartile range, IQR) for each case at each sensor period.^12^ Glucose values were plotted to visualize trends over time, with the hour of day and day of the week on the x-axis. To produce aggregate daily overlay plots, the algorithm rounded each timepoint in the raw data to the nearest 10-min mark and applied LOESS (locally estimated scatterplot smoothing) to generate a smoothed average for each glucose data point at each time of day.

The %TOR >140 mg/dL was calculated for each hour of the day for each sensor wear period and represented in a heatmap. Each heatmap cell displayed percentages on a scale from 0–100%, with higher percentages represented as darker shades. For each sensor, starting at day 1, the numerator for the cumulative %TOR >140 mg/dL was calculated as the sum of each %TOR >140 mg/dL added incrementally. The denominator was the total %TOR >140 mg/dL for the entire wear time across both sensor phases. For each sensor, the cumulative percentage of %TOR >140 mg/dL was then calculated by dividing the numerator by the denominator for each day, starting at day 1. The cumulative percentage of %TOR >140 mg/dL was plotted on the y-axis, with the corresponding day number on the x-axis. The simple slope of cumulative percentage of %TOR >140 mg/dL over time was calculated as the average change in cumulative percentage of %TOR >140 mg/dL for every increase in wear day. Percentage exceeding the median (PEM) was calculated by identifying the median glucose data point in sensor 1 and dividing the number of data points in sensor 2 below the median line by the total number of data points in sensor 2.^13^ The farther the PEM deviates from 50%, the stronger the effect or difference between phases (>75% is a large effect size), signaling a significant change in the pattern of the data. All analyses were conducted in RStudio 4.4.1.

## Results

The participant was a 28-year-old Spanish-speaking Latina with a college degree. Her measured BMI was 30.2 kg/m^2^ (obesity class I), and her CDC Prediabetes Risk Score was 2. She had no current medical conditions and was not currently taking any medications. Eight days of glucose data were available for each sensor, with a 5-day interval between the last day of sensor 1 and the first day of sensor 2. A total of 2,280 sensor readings were captured from sensor 1, and 2,303 sensor readings were captured from sensor 2.

**FIG. 1.**
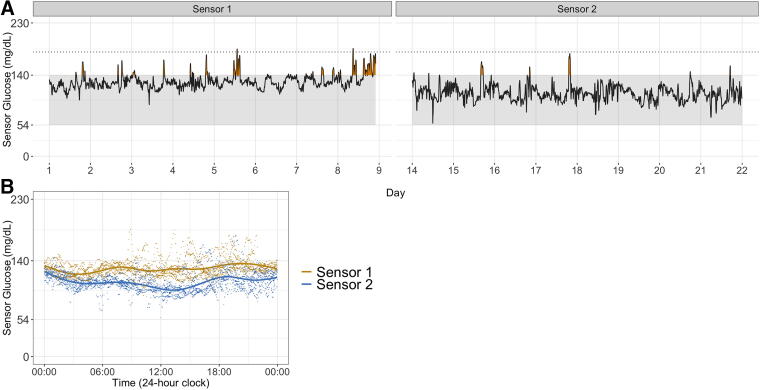
**(A)** Automated record of raw glucose values across all wear days. **(B)** Aggregated daily glucose values by sensor 1 and 2 with LOESS applied. LOESS, locally estimated scatterplot smoothing.

**FIG. 2.**
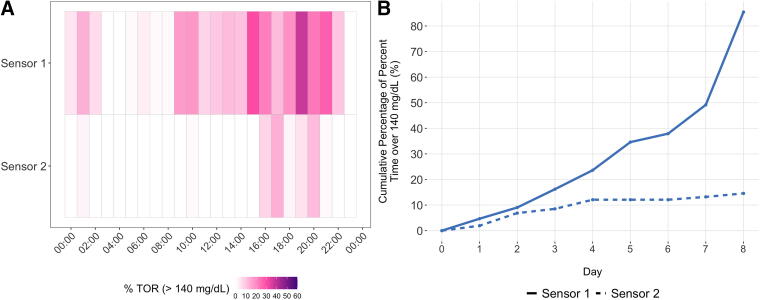
**(A)** Heatmap of %TOR >140 mg/dL aggregated daily by sensor phase. **(B)** Cumulative percentage of %TOR >140 mg/dL sensor glucose. %TOR, percent time out of range.

Figure 1A displays sensor daily glucose across the analytic wear period with a blank gap indicating the non-wear interval between end of sensor 1 and start of sensor 2. The dashed line represents glucose levels >180 mg/dL. Figure 1B shows the aggregated daily sensor glucose overlay LOESS for the first and second wear phase by color. Figure 2A is a heatmap indicating %TOR >140 mg/dL for each hour of the day. Darker colors represent a higher percent. Figure 2B shows the cumulative percentage of %TOR >140 sensor glucose. The denominator is the total %TOR for the total wear time across both wear phases. Over 80% of the cumulative %TOR >140 was recorded ( $\frac{\Delta y}{\Delta x}$ = 10.0) by the first sensor phase, while the second wear phase contributed <20% to cumulative %TOR >140 ( $\frac{\Delta y}{\Delta x}$ = 1.6).

Table 1 shows GV metrics pertinent to people without diabetes. The participant shows a median decrease in %TOR >140 mg/dL in the second wear phase. The number of daily excursions >140 ranged from 0–5 with a median (IQR) of 1.5 (1.0, 2.3) for sensor 1 wear and ranged from 0–1 with a median (IQR) of 0 (0.0, 0.3) for sensor 2 wear. When examining the % excursions >140 mg/dL out of all excursions, there was a reduction for sensor 2. The overall median glucose in sensor 1 was 127 mg/dL and 2,126 data points out of 2,303 were <127 mg/dL in sensor 2, which translates to a PEM of 92.3%, which is a large effect.

**Table 1.** Glycemic Variability (GV) Metrics in Glucose (mg/dL) by Sensor Phase

GV metrics^a^	Sensor 1 (8 days)	Sensor 2 (8 days)
	Median (IQR)	Median (IQR)
Average glucose	126.7 (126.1, 130.3)	108.4 (107.3, 110.5)
%TOR >140	9.2 (5.8, 14.0)	1.9 (1.0, 3.0)
*N* excursions >140	1.5 (1.0, 2.3)	0.0 (0.0, 0.3)
% peaks >140	14.6 (11.8, 20.2)	4.3 (2.8, 5.8)

^a^ All values are median (IQR) in glucose mg/dL.

%TOR, percent time out of range.

## Discussion

This continuous measurement human subject case trial examined glucose in milligrams per deciliter levels in response to a standalone CGM app-based feedback in an adult female with BMI >30 and without diabetes. Our detailed analysis of CGM-generated data and multiple analytic strategies aligns with the growing interest in studying CGM use and effects in people without diabetes. We interpret our results as a step toward providing an account of how CGM feedback can operate in people without diabetes in relation to the suggested %TOR >140 benchmark of <5% daily. The case showed improvement in glycemic control. During the first wear phase, >80% of the total cumulative %TOR >140 was recorded, whereas the second wear period contributed <20% to the total. The cumulative percentage change in %TOR >140 over time, combined with the large effect size detected for PEM at 92%, supports the account that standalone CGM operates on glycemic control when the app was reportedly frequently viewed.

Our case results illustrate the practical utility of CGM as a self-management tool for individuals with obesity as a metabolic risk but without diabetes by offering real-time, personalized feedback that can reinforce behavior change. The CGM system provided continuous glucose data via a smartphone app, including visual cues and auditory alerts for glucose excursions, which allowed the participant to observe the direct effects of dietary behaviors on glucose levels. Over a 16-day period, this standalone feedback mechanism was associated with a marked reduction in time spent above the normoglycemic threshold and a major reduction in daily high-glucose excursions in the second wear phase. The observed improvements in glycemic control, combined with a high percentage of data points falling below the initial median glucose level, suggest that CGM can serve as a behavior-shaping stimulus, enabling users to derive their own *contingency-shaped* rules for modifying behavior within two weeks. These findings support the potential of CGM as a digital health tool for promoting metabolic awareness and facilitating problem-solving strategies relevant to obesity self-management.

Our findings contribute to the growing body of research on CGM use among individuals without diabetes, highlighting its potential role in self-directed obesity management. However, as a case study, the effects observed have limited generality and should be interpreted as preliminary evidence of concept rather than causal proof. Outcomes observed in the single case with college education level may not clearly replicate across cases with differing demographic, digital literacy, and educational histories. Further, unmeasured variables overlapping during the wear period may have operated as artifacts that influenced the observed improvements in glycemic control. Although the participant reported frequent engagement with the CGM app, direct measures of app use were not collected. Future studies should consider incorporating structured behavioral and dietary monitoring to better isolate the contribution of CGM feedback. At the same time, adding these components introduces additional intervention elements that may confound the unique effects of CGM as a standalone feedback tool. There is a long history to the idea of real-time *teaching machines*, and CGM may be the innovation that enables individuals to engage in contingency-shaped problem solving for health-related behavior change.^14^ Given the growing interest in digital health tools, this case study underscores the public health relevance of CGM for supporting metabolic awareness and behavior modification in obesity self-management and diabetes prevention.

## Informed Consent

Informed consent was obtained from all individual participants included in the study.

## Abbreviations Used

CDC: Centers for disease control and prevention
CGM: Continuous glucose monitoring (or continuous glucose monitor, depending on context)
GV: Glycemic variability
HbA1c: Hemoglobin A1c
IQR: Interquartile range
IRB: Institutional review board
LOESS: Locally estimated scatterplot smoothing
NIH: National Institutes of Health
NIMHD: National Institute on Minority Health and Health Disparities
PEM: Percentage exceeding the median
%TOR: Percent time out of range
SCOBe: Systematic coding of observed human behaviour
